# Possibilities and Pitfalls of Social Media for Translational Medicine

**DOI:** 10.3389/fmed.2018.00345

**Published:** 2018-12-06

**Authors:** Suzan Dijkstra, Gautam Kok, Julie G. Ledford, Elena Sandalova, Remi Stevelink

**Affiliations:** ^1^University Medical Center Utrecht, Utrecht, Netherlands; ^2^Department of Cellular and Molecular Medicine, The University of Arizona, Tucson, AZ, United States; ^3^Danone Nutricia Research, Singapore, Singapore; ^4^Department of Pharmaceutical Sciences, Utrecht Institute for Pharmaceutical Sciences, Utrecht University, Utrecht, Netherlands

**Keywords:** translational medicine, translational research, social media, research dissemination, patient engagement, science communication

## Abstract

We live in an age where the sharing of scientific findings and ideas is no longer confined to people with access to academic libraries or scientific journals. Social media have permitted for knowledge and ideas to be shared with an unprecedented speed and magnitude. This has made it possible for research findings to have a greater impact and to be rapidly implemented in society. However, the spread of unfiltered, unreferenced, and non-peer-reviewed articles through social media comes with dangers as well. In this perspective article, we aim to address both the possibilities and pitfalls of social media for translational medicine. We describe how social media can be used for patient engagement, publicity, transparency, sharing of knowledge, and implementing findings in society. Moreover, we warn about the potential pitfalls of social media, which can cause research to be misinterpreted and false beliefs to be spread. We conclude by giving advice on how social media can be harnessed to combat the pitfalls and provide a new avenue for community engagement in translational medicine.

## Introduction

**Figure 1:**
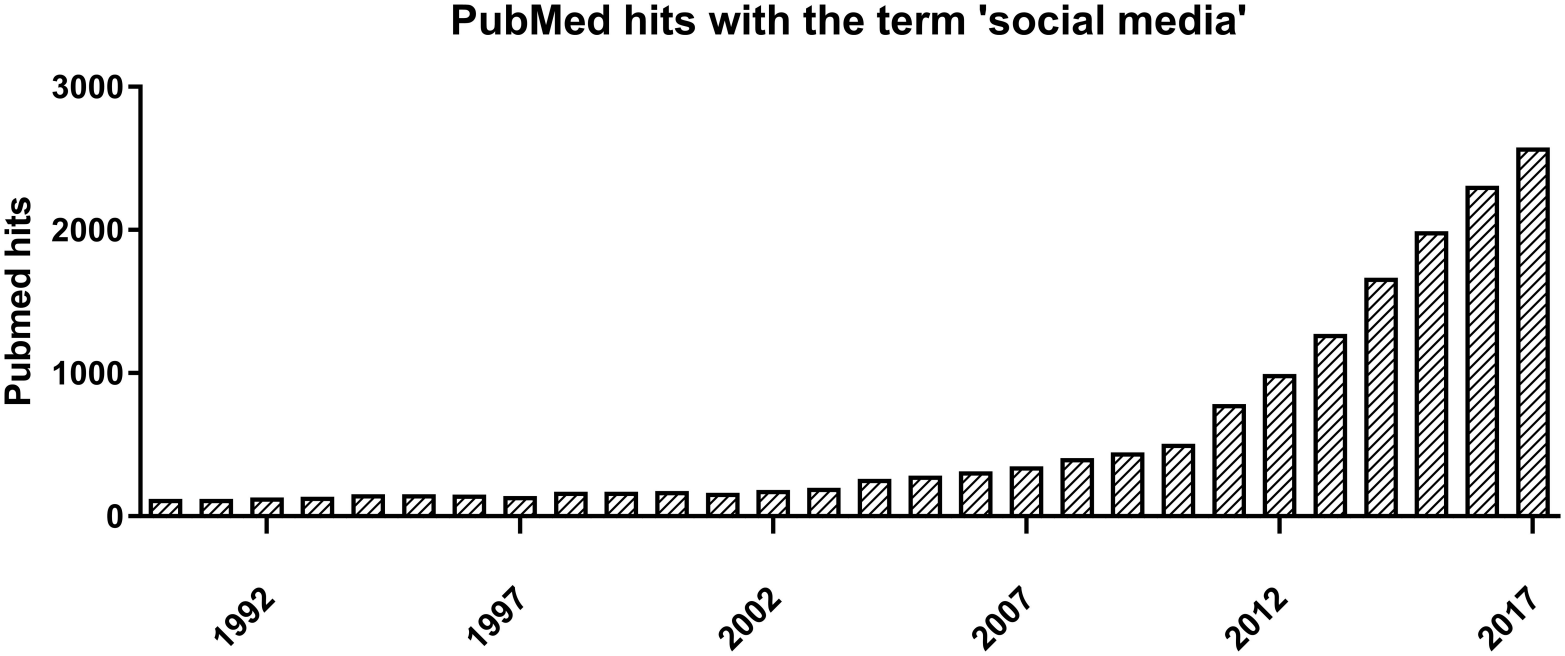
Number of publications found on PubMed with the search term “social media,” as shown by publication year.

The emergence of social media has changed the way we communicate and allows for knowledge and ideas to be shared with an unprecedented speed and magnitude. Similarly, an exponentially increasing amount of research about social media is being published (Figure 1). Social media come in a variety of forms, including collaborative projects such as Wikipedia, (micro)blogs like Twitter, content communities like YouTube, social networking sites like Facebook, and gaming communities like Second Life (1). These platforms are accessible to all and provide forums where people can freely share thoughts, opinions, and knowledge without—in general—any form of censorship or fact-checking.

Several groups have addressed how social media are used by the research and medical communities. Medical researchers have shown doubt about professional use of social media, describing it to be incompatible with research (2). Social media are mostly used for personal and less for professional purposes (3, 4). Yet, on the level of society, social media have great potential. There are many examples of its use for public health and prevention purposes (5, 6). Additionally, the rapid dissemination of research findings and the spreading of knowledge to society has increased public interest and involvement in research. Consequently, patients increasingly can and want to be part of developing solutions for their illness (3, 7).

The use of social media for purposes of implementation and translation of research is still in its early stages. At the same time, social media are clearly being used by both patients and professionals for personal content and information sharing. Various efforts of using social media for research are also increasing. Thus, it is important to raise awareness and understanding of the possibilities and pitfalls that social media present to the research and medical communities as well as to regulatory bodies, patients, and industries. Therefore, in this study, we aimed to address both the possibilities and potential pitfalls of social media for translational medicine. We aimed to provide a brief and broad overview of this topic that could steer the community to be more mindful when using social media. A comprehensive review of all different aspects relating to social media and translational medicine is beyond the scope of this perspective article.

## Possibilities of Social Media for Translational Medicine

### Rapid and Easy Dissemination of Research

Social media are widely used all over the world. Facebook, for example, had an average of 1.45 billion daily active users and 2.20 billion monthly active users in March 2018 (8). With this many users, social media provide platforms for researchers and institutes to quickly disseminate their research plans and findings to a greater public. Through online pages of journals, associations, newsgroups, and direct-sharing, it is relatively easy for researchers to reach a broad audience compared to the more “conventional” sharing of knowledge through publishing in scientific journals. Relevant research findings that are interesting to the community may rapidly spread through social media and go viral. This way, social media may be used to rapidly spread and implement public health findings to the general public. An additional benefit is the easier recruitment of traditionally “hard to reach” populations for medical research (9–11). Furthermore, it increases the chances of research being picked up by peers and stakeholders (4). Faster dissemination of research findings might also prevent other research groups from repeating the same research, decreasing the potential waste of resources. Recently, tools were developed that visualize the magnitude of impact of social media on scientific publications. This is important, as number of tweets within the first 3 days after publication of an article was found to predict which articles would be highly cited on Google Scholar or Scopus (12). The most commonly used tracking tool is Altmetric, which tracks the amount of rumor about an article on nearly all professional and social media outlets (13). For example, an article about the association of fats and carbohydrates with cardiovascular disease published in medical journal *The Lancet* was at time of writing only cited by 21 articles (14). However, the real “buzz” was generated by 8,313 tweets, 450 Facebook posts and 168 news stories, adding it to the top 5% of the most discussed publications of the year (14).

### Critical Review of Existing Articles and Raw Data Sets

In this era of exponentially increasing numbers of publications, using the reviewing power of the scientific community is an opportunity that should not be missed in order to improve overall research quality. As an extension of recent developments toward more transparent peer reviewing, several social platforms that allow open peer review have been developed, encouraging readers to critique existing publications in-depth. In addition, users are stimulated to upload raw data sets as well, including negative results that might otherwise never have been published, thereby counteracting the effect of publication bias (15). However, the scale of impact of open review might be limited to high-profile work that raises concerns, as those are more likely to attract attention (16).

### Possibilities for Raising Funds for Research

With its fast dissemination of information and large number of users, social media platforms have the potential to broadly raise awareness for medical research and specific diseases. Social media platforms have been demonstrated to play an important role in reaching potential donors and raising money in crowd funding campaigns (17). In 2014, $115 million was raised from the *Ice Bucket Challenge* on Facebook for research into new treatment strategies for Amyotrophic Lateral Sclerosis (ALS) (18). In 2016, a 6-year old Dutch boy who was recently diagnosed with a pontine glioma raised € 2.6 million for the Dutch Red Cross by daring people to paint their nails and post a picture on social media (19, 20). Moreover, a social media-based fundraising contest launched by the University of California San Francisco (UCSF) raised more than $1 million for the UCSF Benioff Children's Hospital, surpassing their initial fundraising goal 10-fold (21, 22). Thus, with the large audience that can be reached through social media, new opportunities for raising funds arise.

### Networking Between Clinicians, Researchers, and Patient Groups

Keeping an up-to-date online presence on social media may prove valuable for clinicians and researchers. Social media create an accessible platform for peer-to-peer discussions and form an increasingly important networking tool. Depending on the platform used, potential target audiences include professionals as well as patient representatives.

Social media outlets also enable patients and patient representatives to efficiently unite into groups. This may be especially beneficial for patients with novel or rare diseases (23). In addition to providing guidance, advice, and support to peers, these platforms may be used to exchange and seek medical information from each other and from medical professionals (24). A unique opportunity for clinicians and/or researchers lies in initiating these groups, which facilitates immediate contact with patient groups. This can provide the researcher with valuable first-hand information and enable patients and their representatives to directly influence research and prioritize projects (25). Similar collaborations on social media between patients, clinicians, and researchers have been shown to contribute to overall scientific knowledge (25).

### Big Data Analytics for Prediction Models and Assessing Trends/Outbreaks

Social media outlets have the potential to be used as exponentially growing, observational datasets (26, 27). A well-known example of big data research performed on online data is the prediction of global influenza outbreaks by analyzing the number of searches of the word “influenza” or symptoms of influenza-like illness on Google (also known as *Google Flu Trends*, currently discontinued) (28). The same can be done using data social media such as Twitter. For example, based on data from Twitter posts (tweets) researchers were able to detect increases and decreases in influenza prevalence with a 85% accuracy (29). Another example is a study that found that a model that analyzed language expressed on Twitter was better at predicting atherosclerotic heart disease mortality than a model that combined 10 common risk factors such as smoking, diabetes, and hypertension (30). Social media have also been demonstrated to contain information on health-related behaviors, such as smoking (31), sexual risk behavior (32), and sedentary behavior (33). Finally, they could be used to monitor public opinion on important health topics, such as vaccines (34) and opinions on specific projects or studies (35).

## Potential Pitfalls of Social Media for Translational Medicine

### Lack of Peer Review and Filtering of Quality

The increased speed and magnitude of the spread of scientific findings through social media comes at a price. There is no system for peer review or filtering of social media, which means that any idea can be spread; even if it is fabricated or not supported by evidence. The vast majority of social media users do not have a scientific background and may be ill-equipped to judge the quality of evidence and sources. For example, people might perceive a blog or advertisement stating “proven by science” as just as trustworthy as a research paper in a peer reviewed scientific journal. However, most people will never read the latter; full research articles are simply not as fun and easy to read as readily digestible news items on social media.

### Fake News Spreads Fast and Is Difficult to Refute

Fake news often disseminates rapidly through social media. A recent study compared the differential diffusion of ~126,000 verified true and false news stories through Twitter. Worryingly, the study revealed that false stories spread much faster, further and more broadly than did true news stories. True news stories rarely spread to more than 1,000 people, whereas false stories often reached up to a 100 times more people (36). Similarly, false stories spread several times faster (36), proving what Charles Spurgeon's already asserted in 1855 “a lie will go around the world while truth is pulling its boots on” (37). False stories are generally more novel and trendy than true stories, which are often more sober and nuanced, and it is part of human nature to be attracted to novelty (38). Novel information is most valuable to decision-making (39), and surprising content can induce physiological arousal that encourages people to spread information and cause content to go “viral” (40).

Once a fake story has spread, it becomes increasingly difficult to refute it. This principle is generally known as Brandolini's law, or the “Bullshit Asymmetry Principle”: the amount of energy needed to refute bullshit is an order of magnitude bigger than that needed to produce it (41). Often, the fake news being spread is relatively harmless and primarily amusing. For example, a story by a doctor about a baby boom in Iceland 9 months after a football victory has gone viral, even though it was debunked by statistical analyses (42). Unfortunately, there are also examples of pervasive fake news stories that endanger public health. Perhaps the most famous of these stories is the case of Dr. Wakefield, who wrote an article that suggested a link between the MMR-vaccine and autism (43). The study was soon discovered to be fraudulent, the article was officially retracted, and Dr. Wakefield's UK medical license was retracted (44). It is now 14 years after the retraction of this article, but its fraudulent results continue to refrain people from taking vaccinations (45). A search on Facebook reveals 109 public pages and 94 discussion groups about vaccines with collectively more than a million members and followers, such as @*thetruthaboutvaccines* (136 k followers) where daily memes are posted to warn people about putative risks of vaccination, including autism. Psychological studies have shown that incorrect memories continue to influence decision making even when you are aware that the memory is false (46), which may explain part of the persistence of these stories. Similarly, most strategies to correct vaccine misinformation are ineffective and could even backfire (47). With fake news being this difficult to refute, it invites the question whether the dangers of the fast and broad dissemination on social media outweigh the advantages.

### Misinterpretation of Research

Aside from fake or fraudulent research being spread on social media, there is also the risk of genuine research findings to be misinterpreted. Conclusions of research findings are often simplified and overly extrapolated in the media. A prime example of this happened in 2015, when a study on cancer risk was published (48). The authors concluded that 65% of the variation in cancer risk among different tissues could be explained by the total number of stem cell divisions and thus “bad luck” (i.e., random mutations arising during DNA replication in normal, non-cancerous stem cells). Even though the study did not explore the causes of cancer, major news headlines (mis)interpreted: “most cancers are caused by bad luck–not bad judgement, says study” (49), “most cancers are ‘caused by bad luck–not lifestyle”” (50), and similar titles (51). Six days after publication, an additional press release addressed these erroneous conclusions, but they had already been shared on social media extensively. This exemplifies the damage that can be done when research findings are misinterpreted and spread to the general public.

### Dissemination of Pseudoscience Through Social Media

The line between science and pseudoscience is often blurred and it is difficult to determine what is true and false (52, 53). Sometimes, pseudoscientific information can give false hope to patients with disease. Moreover, while pseudoscientific supplements are often relatively harmless, there are also dangerous advices and practices, which are readily being spread through social media. For example, the use of alternative treatments and supplements without proven efficacy (52) are often promoted through social media. Moreover, multiple procedures for tampering with existing drugs can be obtained via the internet (53). These procedures are illegal and unconfirmed to result in the drug formulation of interest, which in some cases can even lead to (fatal) intoxications (54). This makes the spreading of pseudoscientific findings a potentially harmful situation.

With the increased use of social media, the public is paying closer attention to bloggers and celebrities—regardless of their medical or scientific background—than to experts in their respective fields of interest. For example, Dr. Mercola, an osteopathic physician, has almost 2 million followers on Facebook, a strong online presence and daily emails to subscribers where he pushes “alternative” or “miracle” supplements to the masses. However, in 2016, Dr. Mercola, was ordered to refund customers up to $5.3 million for the false advertisement of his own company's tanning beds that he claimed would reduce chances of getting cancer. This was not his first trouble with regulators: the US Food and Drug Administration (FDA) warned him three times between 2005 and 2011 for violating federal laws for marketing a device he claimed was an alternative to mammograms and for making unproven claims about dietary supplements (55). Dr. Oz is another proponent of pseudoscience and “miracle cures” for an array of conditions. He has 6 million Facebook followers and his own television show. Perhaps most notable is his persistent advertising of “miracle” weight loss supplements that will be effective with little to no exercise. He was criticized by the Senate in 2014 for such unsupported claims for specific supplements and was called to be removed from the faculty at Columbia University, where he worked as a cardiothoracic surgeon. During his testimony, Dr. Oz acknowledged that many supplements he lends support to would not stand up to scientific scrutiny (56) and a recent study confirmed that most of his claims were not supported and, in some instances, contradicted by evidence (57). These instances are just the tip of the iceberg when it comes to examples of pseudoscientific ideas being spread to a large audience.

## How to Best Use Social Media

In 2016, politician Michael Gove famously claimed “people have had enough of experts” (58). This assertion was confirmed when the majority of the UK voted to leave the EU against all expert advice. What does this mean for us as a research community, the “experts” on healthcare, and how can we use social media to combat fake news and pseudoscience that could endanger translational medicine and public health?

We believe that we, as a research community, have a responsibility to use social media to spread research findings of public interest and to combat fake news that can be harmful to society. One way to counter the dangerous spread of misinformation is for scientists to critically evaluate the scientific news stories and report inaccuracies in order to correct or refute them. As news media outlets are more likely to report data that are compelling or sensational, it is essential to provide information that is interesting to the general public while at same time maintaining standards for reporting the accuracy of the relayed information (59). Another possibility is for the scientific community to use a rating and online review system similar to travel-review websites such as TripAdvisor, in order to establish consensus about the validity and quality of research and health claims that are circulating on the internet (41). Moreover, several social media groups have been established specifically for refuting false news, such as the Facebook and Twitter group “Refutations to Anti-Vaccine Memes” (@*RtAVM*), which has 233,871 members that aim to refute fake news stories about anti-vaccine movements by responding with rational arguments and counter-memes that dispel false-beliefs. However, confirmation bias can be strong and it remains to be seen whether people with opposing views will be convinced or even read such pages with opposing views.

Another approach for scientists to reach people with opposing views is to think small and to begin with sharing information within their immediate social network. Many scientists have several hundreds of social media connections, 519 on average, and these personal connections could mean that people trust and value their opinions, especially in their field. It has been suggested that every scientist can be a “nerd of trust” within their network of friends and family, and collectively, we as a scientific community could have the potential to influence the opinion of a large part of society (60).

## Conclusion

We live in an exciting age, where social media allow for unrestricted spreading of scientific findings at an extraordinary pace, which brings major advantages for translational medicine, but comes with several potential dangers and pitfalls as well (as summarized in Table 1). We hope that this perspective article helps translational researchers to tackle the challenges and harness the possibilities of social media for the advancement of science.

**Table 1:** Possibilities and pitfalls of social media use for translational medicine.

	**Possibilities**	**Pitfalls**
1	Rapid and easy dissemination of research	Lack of peer review and filtering of quality
2	Critical review of existing articles and raw data sets	Fake news spreads fast and is difficult to refute
3	Possibilities for raising funds for research	Misinterpretation of research
4	Publicity of researchers/institutes	Dissemination of pseudoscience through social media
5	Networking between clinicians, researchers and patient groups
6	Big data analytics for prediction models and assessing trends/outbreaks

## Author Contributions

SD, GK, JL, ES and RS contributed to the conceptualization and writing of this study. SD, GK, and RS wrote and edited the final manuscript. RS coordinated the study. SD and GK contributed equally to this work.

### Conflict of Interest Statement

ES is employed by Danone Nutricia Research. The remaining authors declare that the research was conducted in the absence of any commercial or financial relationships that could be construed as a potential conflict of interest.
